# Comparative Transcriptome Profiling Reveals Changes of microRNAs Response to Exercise in Rats with Neuropathic Pain

**DOI:** 10.1155/2021/5597139

**Published:** 2021-08-02

**Authors:** Jia-Bao Guo, Bing-Lin Chen, Ge Song, Yi-Li Zheng, Yi Zhu, Zheng Yang, Xuan Su, Ying Wang, Qing Cao, Pei-Jie Chen, Xue-Qiang Wang

**Affiliations:** ^1^The Second Clinical Medical College, Xuzhou Medical University, Xuzhou, Jiangsu, China; ^2^Department of Sport Rehabilitation, Shanghai University of Sport, Shanghai, China; ^3^The Fifth Affiliated Hospital of Zhengzhou University, Zhengzhou, Henan, China

## Abstract

There is accumulating evidence showing that exercise therapy may play an active role in peripheral neuropathic pain (NP), but its mechanism is still unclear. Studies have found that microRNAs (miRNAs) may play a role in NP by regulating pain-related target genes. Therefore, we aimed to explore the changes of miRNA and mRNA of dorsal root ganglion (DRG) after NP in response to exercise with transcriptome technology. The chronic constriction injury (CCI) model was established, and rats were randomly allocated into three groups, namely, the sham-operated, CCI, and CCI-exercised groups. L4-L6 DRG tissue was taken for RNA-sequencing, and the differentially expressed genes (DEGs) were determined through bioinformatics analysis. Real-time PCR was used to confirm the accuracy. A total of 4 overlapping differentially expressed miRNAs and 186 overlapping differentially expressed mRNAs were identified in the two comparisons of the sham-operated group versus the CCI group and the CCI group versus the CCI-exercised group. Among these DEGs, miR-145-5p, miR-341, miR-300-5p, miR-653-5p, Atf3, Cacna2d1, Gal, and Ctss related to NP were validated by real-time PCR. DEGs between the CCI and CCI-exercised groups were enriched in HIF-1 signaling pathway, Rap1 signaling pathway, and neurotrophin signaling pathway. This study provides an understanding of the adaptive mechanisms after exercise of NP, and these DEGs in DRG might play a role in NP by stimulating the enriched pathways.

## 1. Introduction

Neuropathic pain (NP) is an unpleasant sensory and emotional experience caused by a lesion of or disease to the somatosensory system [1]. NP is typified by a multitude of signs, including spontaneous pain, allodynia, hyperalgesia, and paraesthesia. Unlike nociceptive pain, NP has poor response to standard analgesics; indeed, only 33%–50% patients benefit from first-line analgesics [2]. A previous report revealed that 17% of NP patients scored its impact on their quality of life as “worse than death” [3]. Thus, we must better understand NP in order to develop effective therapeutics.

Exercise has become an increasingly popular nonpharmacological approach to treat NP. Some evidence that exercise can be safe and beneficial for pain management for NP has been observed in a population of peripheral NP patients [4–7]. Aerobic exercise can increase motor conduction velocity and epidermal innervation and decrease pain ratings in people with diabetic peripheral neuropathy [8, 9]. However, although the mechanism of exercise training in improving NP induced by peripheral nerve injury has been discussed in some preclinical rodent studies, such as by decreasing proinflammatory cytokine expression [10–12], the molecular mechanisms triggering and maintaining these benefits remain poorly understood.

Gene expression profile research can help researchers better understand the mechanisms of NP in response to exercise. Microarray or RNA-sequencing (RNA-seq) technology has been implemented to investigate the differentially expressed genes (DEGs) in the comparison of sham-operated rats versus NP rats [13–15]. DEG analysis can help discover quantitative changes in expression levels between experimental groups by using statistical analysis [16]. However, no study assessing the effects of exercise on dorsal root ganglion (DRG) transcriptome in NP model is yet available. In the present paper, RNA-seq was utilized to reveal the molecular responses of chronic constriction injury (CCI) rats to exercise, and DEGs and their functional associations were analyzed.

## 2. Methods

### 2.1. Animals and Groups

Adult male Sprague–Dawley rats (RRID: RGD_70508) in the weight range of 200 g to 220 g (6 weeks old) were purchased from Shanghai SLAC Laboratory. The animals were housed five to a cage with free access to food and water, and the room was maintained at a temperature of 24 ± 1°C under a 12 : 12 h dark–light cycle. All animal procedures have been approved by the Animal Care and Use Committee of Shanghai University of Sport (No. 2018006).

**Figure 1:**
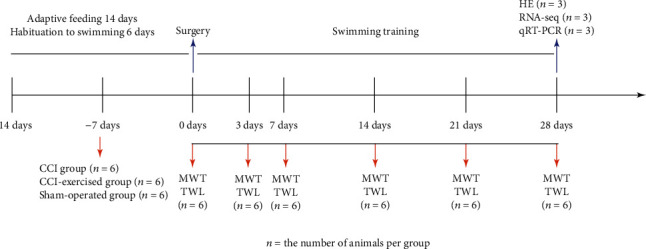
Experimental study design. The arrows indicate where behavioral tests were performed or samples were obtained for endpoint analysis. MWT: mechanical withdrawal threshold; TWL: thermal withdrawal latency; HE: hematoxylin-eosin staining; RNA-seq: RNA-sequencing; qRT-PCR: quantitative real-time PCR.

After 7 days of adaptive feeding, the animals were randomly allocated into three groups: rats with CCI (CCI group, *n* = 6), rats with CCI that received swimming treatment (CCI-exercised group, *n* = 6), and rats with sham operation (sham-operated group, *n* = 6). The CCI group and sham-operated group did not receive swimming treatment. Details regarding the experimental grouping and number of animals used in each part are shown in Figure 1.

### 2.2. Chronic Constriction Injury Models

The CCI model was constructed according to a previous description [17]. Briefly, the rats were first anaesthetized in the induction chamber with 5% isoflurane in O_2_ and then administered 2% isoflurane in O_2_ by using a facemask. The right hind leg of each rat was shaved and sterilized before surgery. An incision was made into the skin 3–4 mm below and parallel to the femur bone. The sciatic nerve of the right hind limb was exposed after performing blunt separation for connective tissues between the gluteus superficialis and the biceps femoris muscles. We used 4–0 chromic catgut to tie four loose ligatures around the exposed sciatic nerve at distances of 1 mm apart as described by Bennett and Xie [17]. Skin incisions were closed with 5–0 silk sutures. Sham operations involved an identical procedure to expose the sciatic nerve, but the nerve was not ligated. No additional medications or analgesics were given to the animals after surgery to reduce pain as this study observed outcomes related to pain.

### 2.3. Exercise Protocols

**Figure 2:**
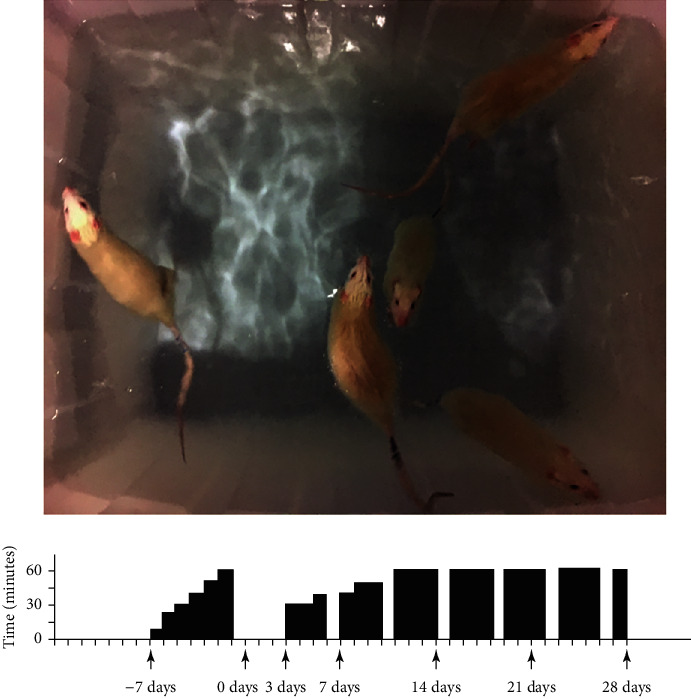
Protocol for swimming training. The training program involves one week of habituation to swimming before surgery and 4 weeks of formal swimming training after surgery. The black bar represents the time of training every day, and if there is no black bar, it means rest on that day.

The protocol for swimming training is shown in Figure 2 and involved 6 days of habituation to swimming before surgery and 28 days of swimming training after surgery [18, 19]. Animals were placed in a plastic container (length = 82 cm, width = 60 cm, and height = 59 cm) filled with approximately 200 L of water (37°C). The training process was supervised by researchers to avoid floating behavior. Once floating behavior was observed, the researchers would nudge the nape of the rat with a pen or stir the water to create a current [20, 21]. After exercise, the rats were gently dried with a cloth towel.

### 2.4. Behavioral Testing

Behavioral testing was conducted before surgery and on the 3rd, 7th, 14th, 21st, and 28th postoperative day. The rats were habituated in individual glass chambers (11 cm × 22 cm × 13 cm) on a wire mesh table for 20 minutes before each test.

#### 2.4.1. Mechanical Withdrawal Threshold (MWT)

To determine the mechanical sensitivity of the hind paw, MWT was measured by using Von Frey filaments (Aesthesio, Danmic Global, USA). The test area was the midplantar surface of the hind paw, and the force of the Von Frey hairs was increased until the expected responses were induced. The range of stimulus forces was 4–180 g. Each filament from small to large was used to stimulate five times, and the force at which the rat withdrew the paw at least three times was recorded as MWT [22].

#### 2.4.2. Thermal Withdrawal Latency (TWL)

A thermal plantar algesimeter (Cat. No. 37370, Ugo Basile, Italy) was utilized to test thermal sensitivity. The rats were put on the plexiglass floor of the device with the infrared source directly beneath it. The midplantar surface of the hind paw was exposed to a radiant heat source with an intensity of 20 I.R., and a cutoff time of 30 seconds was used to protect rats from tissue injury. The latency of withdrawal was measured in seconds. This step was repeated five times at 5 min intervals between stimuli, and then, the mean TWL was calculated.

### 2.5. Hematoxylin-Eosin (HE) Staining

Three rats were randomly selected from each group for this analysis. The rats were deeply anesthetized with 3% pentobarbital sodium via intraperitoneal (i.p.) injection (40 mg/kg) 28 days after surgery to reduce the suffering before perfusion. In addition, the animal procedures were performed by experienced experimenters to ensure that no extra pain was caused to the rats during the experiments. The rats were perfused with 250 mL of normal saline (4°C) through the ascending aorta followed by 200 mL of 4% paraformaldehyde solution (4°C). Then, a 1 cm length of the injured sciatic nerve was obtained and postfixed in 4% paraformaldehyde solution for 90 minutes at 4°C. After postfixation, sciatic nerves were transferred to 15% sucrose solution for at least 24 hours and then transferred to 30% sucrose solution for at least 48 hours. Fixed tissues were embedded in Tissue Freezing Medium (Cat. No. 4583, SAKURA, CA, USA) and cryosectioned at 14 *μ*m sections on a Leica CM1950 cryostat. The sections were visualized using an Olympus BX53 microscope (Tokyo, Japan) and images captured using Cell Sens Dimension software (version 1.15; Olympus; RRID:SCR_016238) with an Olympus DP80 digital camera (Olympus, Tokyo, Japan) attached to the microscope.

### 2.6. RNA-seq

For RNA-seq analysis, the remaining three rats were deeply anesthetized with 3% pentobarbital sodium via intraperitoneal (i.p.) injection (40 mg/kg) 28 days after surgery and perfused with 250 mL of normal saline (4°C) through the ascending aorta, and tissues of L4–L6 DRG were collected. Total RNA was extracted from the tissues using a miRNeasy Micro Kit (Cat. No. 217084, Qiagen, Hilden, Germany). Strict quality control of the RNA samples after RNA extraction included the following aspects: agarose gel electrophoresis to test RNA degradation and contamination, NanoPhotometer® spectrophotometry (IMPLEN, CA, USA) to assess the purity of RNA, Qubit® RNA Assay Kit (Cat. No. Q10210, Thermo Fisher Scientific, MA, USA) in Qubit® 2.0 Fluorometer (Life Technologies, CA, USA) to measure RNA concentrations, and the RNA Nano 6000 Assay Kit of the Bioanalyzer 2100 system (Cat. No. 5067-1511, Agilent Technologies, CA, USA) to determine RNA integrity. After the RNA samples met the quality requirements, the NEBNext® Multiplex Small RNA Library Prep Set for Illumina® (Cat. No. E7300L, NEB, MA, USA) and NEBNext® UltraTM RNA Library Prep Kit for Illumina® (Cat. No. E7530L, NEB, MA, USA) were utilized to create sequencing libraries. Finally, the library preparation was sequenced on the Illumina Hiseq 2500/2000 platform.

### 2.7. Bioinformatics Analysis

First, the quality of raw data obtained from RNA-seq was evaluated, including filtering the raw data, checking the error rate, and GC content distribution. Then after mapping, assembly was done followed by quantification. To analyze the RNA-seq data, the DESeq2 R package (RRID: SCR_015687) was utilized to calculate DEGs in the two comparisons (sham-operated group versus CCI group and CCI group versus CCI-exercised group) and request the *p* values less than 0.05. The miRNA target genes were predicted as the intersection of the two software, miRanda and RNAhybrid. We then cross the predicted miRNA target genes with the mRNA-seq results to obtain their intersection genes. Gene Ontology (GO; RRID: SCR_002143) enrichment analysis was used on the DEGs by GOseq software (RRID: SCR_017052). Kyoto Encyclopedia of Genes and Genomes (KEGG) pathway analysis (RRID: SCR_012773) was implemented to understand the functions and utility of the biological system identified (http://www.genome.jp/kegg/) [23], and KEGG Orthology-Based Annotation System software (RRID: SCR_006350) was utilized to identify the enriched pathways [24]. *p* values less than 0.05 were set as enrichened significance.

### 2.8. Quantitative Real-Time PCR (qRT-PCR) Analysis

The DEGs were validated through qRT-PCR of the nine sequenced samples to identify the accuracy of the RNA-seq results. The first-strand cDNA was synthesized by using the miScript II RT Kit (Cat. No. 218161, Qiagen, Hilden, Germany). Then, the first-strand cDNA was amplified by using the miScript® SYBR® Green PCR Kit (Cat. No. 218073, Qiagen, Hilden, Germany) and QuantiNova SYBR Green PCR Kit (Cat. No. 208054, Qiagen, Hilden, Germany) with a real-time PCR system (Applied Biosystems StepOnePlus, CA, USA). The primers used are provided in Table 1. The comparative Ct method (*ΔΔ*Ct) was used to quantify the expression of these miRNAs and mRNAs, and relative expressions were normalized to that of U6 and 18S RNA by using the 2^−*ΔΔ*Ct^ method.

### 2.9. Statistical Analysis

All statistical calculations were performed using SPSS software (version 16.0, SPSS, Inc., Chicago, IL, USA; RRID: SCR_002865). Data of the characteristics of each group are expressed as the mean ± standard error of mean (SEM). Results from the behavioral tests were analyzed using two-way repeated-measure analysis of variance (ANOVA). In addition, results of qRT-PCR were analyzed using one-way ANOVA followed by Tukey's multiple comparison test. *p* values less than 0.05 were set as statistical significance.

## 3. Results

### 3.1. Animal Characteristics

**Figure 3:**
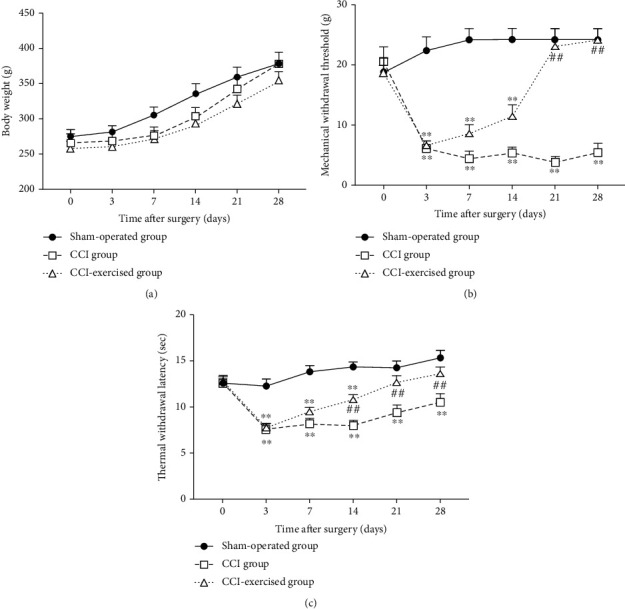
Body weight and nociceptive behavior in different time courses. (a) Time courses of body weight, (b) mechanical withdrawal threshold, and (c) thermal withdrawal latency. Analyses of data were done by two-way ANOVA for repeated measures. Values indicate the mean ± standard error of mean (*n* = 6 animals/group). ^∗^*p* < 0.05 and ^∗∗^*p* < 0.01, for comparisons of the sham-operated group vs. the chronic constrictive injury (CCI) group or CCI-exercised group. ^#^*p* < 0.05 and ^##^*p* < 0.01 for comparisons of the CCI-exercised group vs. the CCI group.

The characteristics of the three groups are illustrated in Figure 3. Body weight, MWT, and TWL were measured 0, 3, 7, 14, and 28 days after surgery. The CCI and CCI-exercised groups showed no significant difference in body weight when compared with the sham group (Figure 3(a)). Results also demonstrated that the MWTs of the CCI and CCI-exercised groups were significantly lower than that of the sham-operated group on the 3rd (*p* < 0.01), 7th (*p* < 0.01), and 14th (*p* < 0.01) postoperative days (Figure 3(b)). On the 21st (*p* < 0.01) and 28th (*p* < 0.01) postoperative days, the level of MWT in the CCI-exercised group was significantly increased compared with that in the CCI group. Furthermore, no significant difference was observed between rats in the sham and CCI-exercised groups on the 21st and 28th postoperative days. In contrast to exercised rats, MWT was significantly reduced in CCI rats (*p* < 0.01). The results presented in Figure 3(c) show that the TWL, CCI, and CCI-exercised groups indicated reduced thermal hypersensitivity on postoperative days 3 (*p* < 0.01), 7 (*p* < 0.01), and 14 (*p* < 0.01) compared with the sham-operated group. Similarly, swimming increased TWL in the CCI-exercised group on days 14 (*p* < 0.01), 21 (*p* < 0.01), and 28 (*p* < 0.01) compared with that of the CCI group.

### 3.2. Histopathology Analysis of Sciatic Nerves

**Figure 4:**
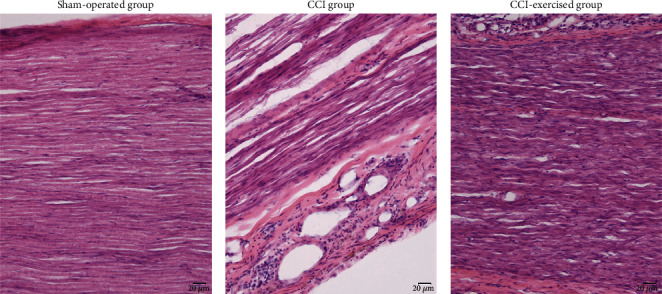
Histological changes of sciatic nerves. The hematoxylin-eosin (HE) sections were observed under light microscopy (×200, bar = 20 *μ*m).

HE staining was used to observe the histopathological changes of the sciatic nerve in each group, further confirming whether the establishment of the CCI model was successful. As shown in Figure 4, the HE sections showed that sciatic nerve fibers were scattered, myelin sheath vacuolated, Schwann cells proliferated, and peripheral inflammatory cells infiltrated in the CCI group, which indicated the success of the CCI model. The morphology of nerve fibers, proliferation of Schwann cells, and infiltration of peripheral inflammatory cells in CCI exercise group were significantly improved, which was similar to that in the sham-operated group.

### 3.3. Identification of Differentially Expressed Genes

**Figure 5:**
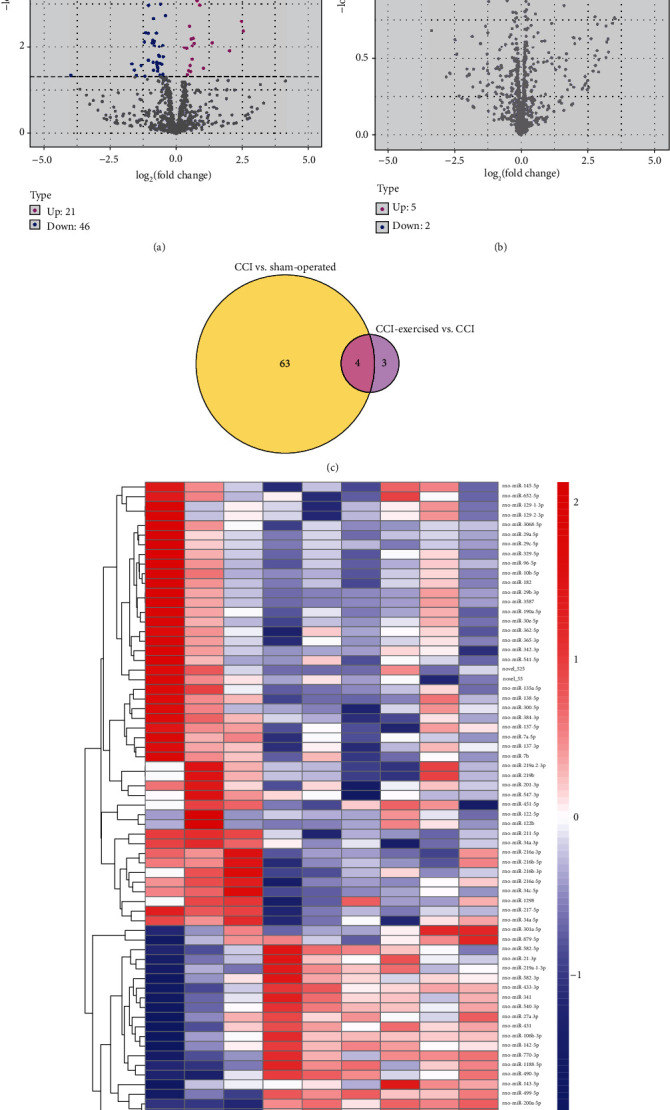
Expression profile changes in microRNAs (miRNAs) in the dorsal root ganglion (DRG). (a) Volcano plots of RNA-sequencing data showing differentially expressed miRNAs between the chronic constrictive injury (CCI) and sham-operated groups. (b) Volcano plot showing differentially expressed miRNAs between the CCI and CCI-exercised groups. Magenta dots represent genes with significantly upregulated expression, blue dots represent genes with significantly downregulated expression, and grey dots represent genes with no significant difference. (c) Overlap of differentially expressed miRNAs between the two comparisons shown as a Venn diagram. (d) Heatmap showing the relative expression levels of differentially expressed miRNAs among the three groups. Upregulated and downregulated genes are colored red and blue, respectively (*n* = 3 animals per group).

The optical density 260/280 of all RNA samples ranged from 1.9 to 2.2, and the number of RNA integrity was greater than 7. The quality of raw data was assessed, and the results are shown in Tables S1–S2.  Figure 5 shows the results of differentially expressed miRNAs in the two comparisons (sham-operated group versus CCI group and CCI group versus CCI-exercised group). Among the identified differentially expressed miRNAs, 67 genes were found to be differentially expressed between the sham-operated and CCI groups, with 21 upregulated and 46 downregulated miRNAs (Figure 5(a)). After 4 weeks of swimming, 7 miRNAs with significantly changed expression were observed in CCI-exercised rats compared with CCI rats. Among these miRNAs, 5 and 2 miRNAs were upregulated and downregulated (Figure 5(b)), respectively. Four overlapping differentially expressed miRNAs (miR-145-5p, miR-341, miR-300-5p, and miR-653-5p) were identified in a Venn diagram (Figure 5(c)). Hierarchical clustering analysis of differentially expressed miRNAs was used to determine the clustering pattern of the three groups (Figure 5(d)).

**Figure 6:**
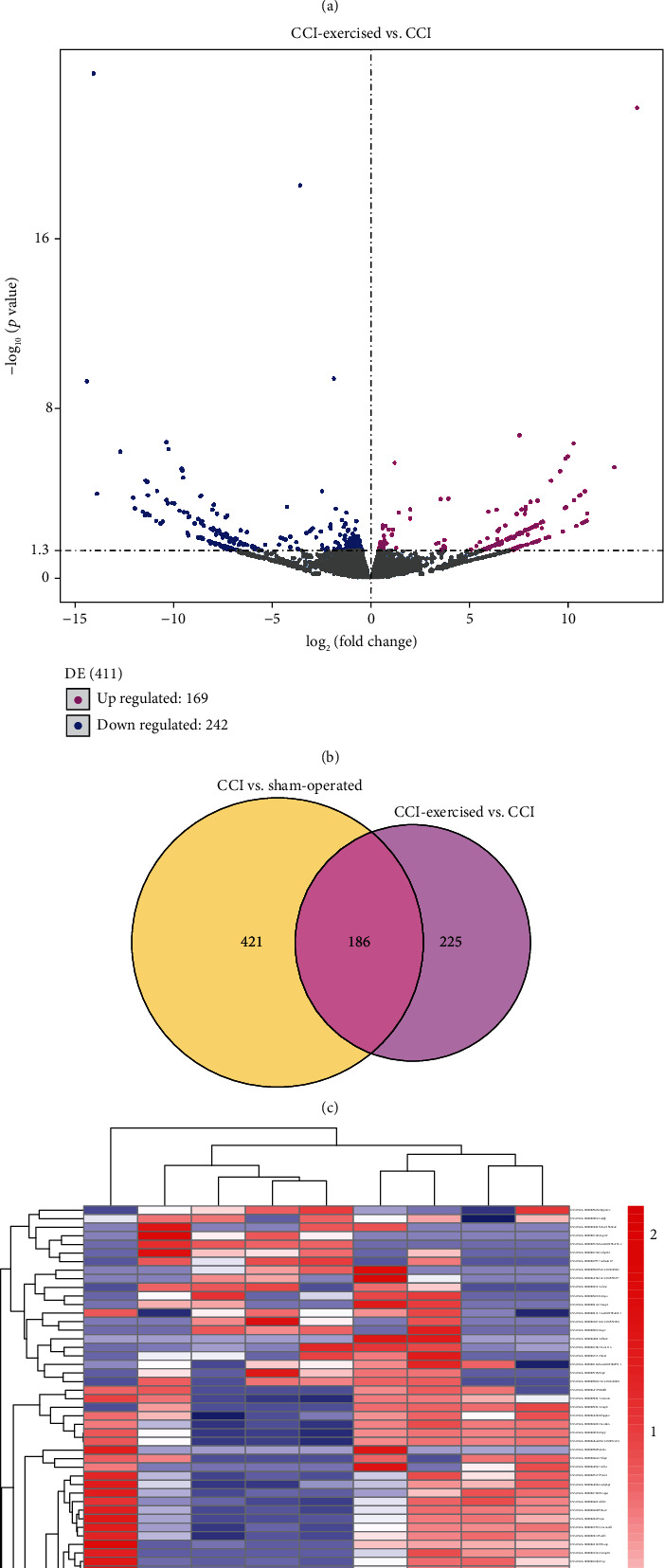
Expression profile changes in mRNA in the dorsal root ganglion (DRG). (a) Volcano plot indicating upregulated and downregulated differentially expressed mRNAs between the chronic constrictive injury (CCI) and sham-operated groups. (b) Volcano plot indicating upregulated and downregulated differentially expressed mRNAs between the CCI and CCI-exercised groups. Magenta dots represent genes with significantly upregulated expression, blue dots represent genes with significantly downregulated expression, and grey dots represent genes with no significant difference. (c) Overlap of differentially expressed mRNAs between the two comparisons shown as a Venn diagram. (d) Heatmap showing the relative expression levels of differentially expressed mRNAs among the three groups. Upregulated and downregulated genes are colored red and blue, respectively (*n* = 3 animals per group).

In addition, Figure 6 shows the results of differentially expressed mRNAs. The volcano plot in Figure 6(a) indicates that 363 and 244 mRNAs are upregulated and downregulated, respectively, between the CCI and sham groups. After 4 weeks of swimming, 169 and 242 mRNAs were upregulated and downregulated, respectively, as observed in Figure 6(b). A total of 186 overlapping differentially expressed mRNAs were found in Figure 6(c). Among 186 differentially expressed mRNAs, 14 mRNAs were identified to be related to NP and multiple-related phenotype by searching the Rat Genome Database (RGD, Table 2). According to the disease annotations in RGD, 6, 4, 2, 6, and 6 mRNAs were associated with nervous system disease, neuralgia, pain, hyperalgesia, and inflammation, respectively. Hierarchical clustering analysis of differentially expressed mRNA was used to show the clustering pattern of the differential mRNA expression of the three groups (Figure 6(d)).

Two softwares are used to predict target genes in overlapping differentially expressed miRNAs (miR-145-5p, miR-341, miR-300-5p, and miR-653-5p), and the result is that a total of 125 target genes can be predicted by the four miRNAs. Then, it was crossed with the sequencing results of 186 overlapping differentially expressed mRNAs to obtain a total of 1 cross gene (Lrguk).

### 3.4. GO Analysis of DEGs

**Figure 7:**
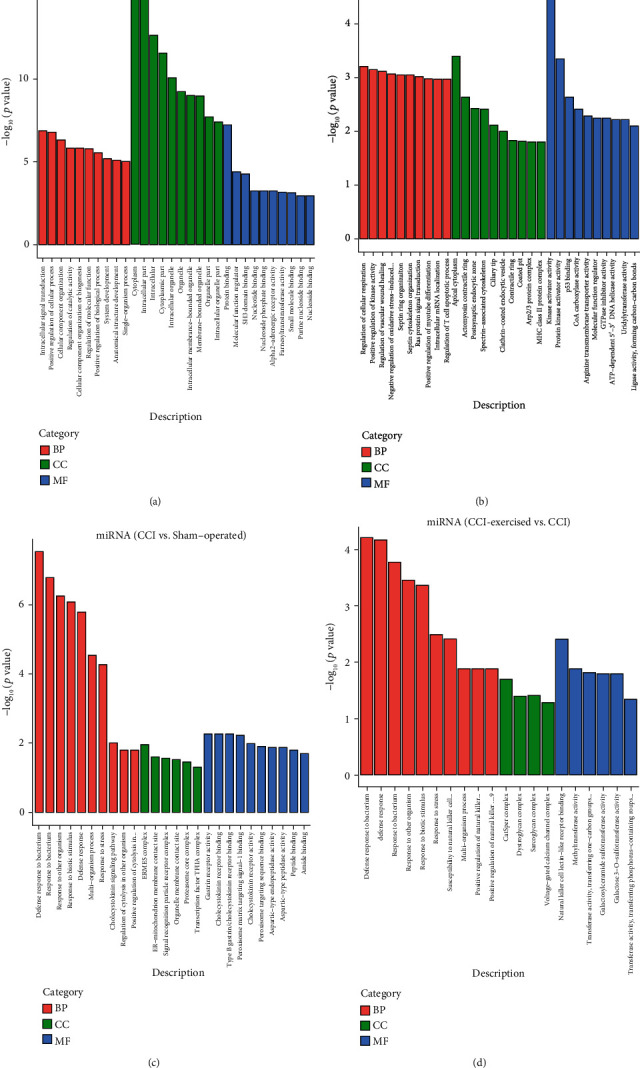
Gene Ontology (GO) analysis of differentially expressed genes (DRGs). The top 10 high-enrichment score terms of differentially expressed microRNAs (miRNAs) are shown in the histography (a) between the sham-operated group and the chronic constrictive injury (CCI) group and (b) between the CCI group and the CCI-exercised group. The top 10 high-enrichment score terms of differentially expressed mRNAs are shown in the histography (c) between the sham-operated group and the CCI group and (d) between the CCI group and the CCI-exercised group (*n* = 3 animals per group).

According to the target gene candidates of differentially expressed miRNAs, GO analysis was performed to explore the biological process (BP), cellular component (CC), and molecular function (MF) of DEGs in the two comparisons (sham-operated group versus CCI group and CCI group versus CCI-exercised group).  Figure 7(a) shows the top 10 GO terms in the comparison between the sham-operated and CCI groups (*p* < 0.05). The most significantly enriched BPs were intracellular signal transduction, positive regulation of cellular process, and cellular component organization. The most significantly enriched CCs were cytoplasm, intracellular part, and intracellular. The most significantly enriched MFs were protein binding, MF regulator, and SH3 domain binding. The top 10 GO terms in the comparison between the CCI and CCI-exercised groups are shown in Figure 7(b). The most significantly enriched BPs were regulation of cellular respiration, positive regulation of kinase activity, and regulation of vascular wound healing. The most significantly enriched CCs were apical cytoplasm, actomyosin contractile ring, and postsynaptic endocytic zone. The most significantly enriched MFs were kinase activator activity, protein kinase activator activity, and p53 binding.

The enriched GO terms of differentially expressed mRNAs between the two comparisons are shown in Figures 7(c) and 7(d) (*p* < 0.05). Between the sham-operated and CCI groups, the most significantly enriched BPs were defense response to bacterium, response to bacterium, and response to another organism. The most enriched CCs were ERMES complex, ER–mitochondrion membrane contact site, and signal recognition particle receptor complex. The most significantly enriched MFs were gastrin receptor activity, cholecystokinin receptor binding, and type B gastrin/cholecystokinin receptor binding (Figure 7(c)). Between the CCI and CCI-exercised groups, the most significantly enriched BPs were defense response to bacterium, defense response, and response to bacterium. The most enriched CCs involved CatSper, dystroglycan, and sarcoglycan complexes. The most significantly enriched MFs were natural killer cell lectin-like receptor binding, methyltransferase activity, and transferase activity (Figure 7(d)).

### 3.5. KEGG Pathway Analysis of DEGs

**Figure 8:**
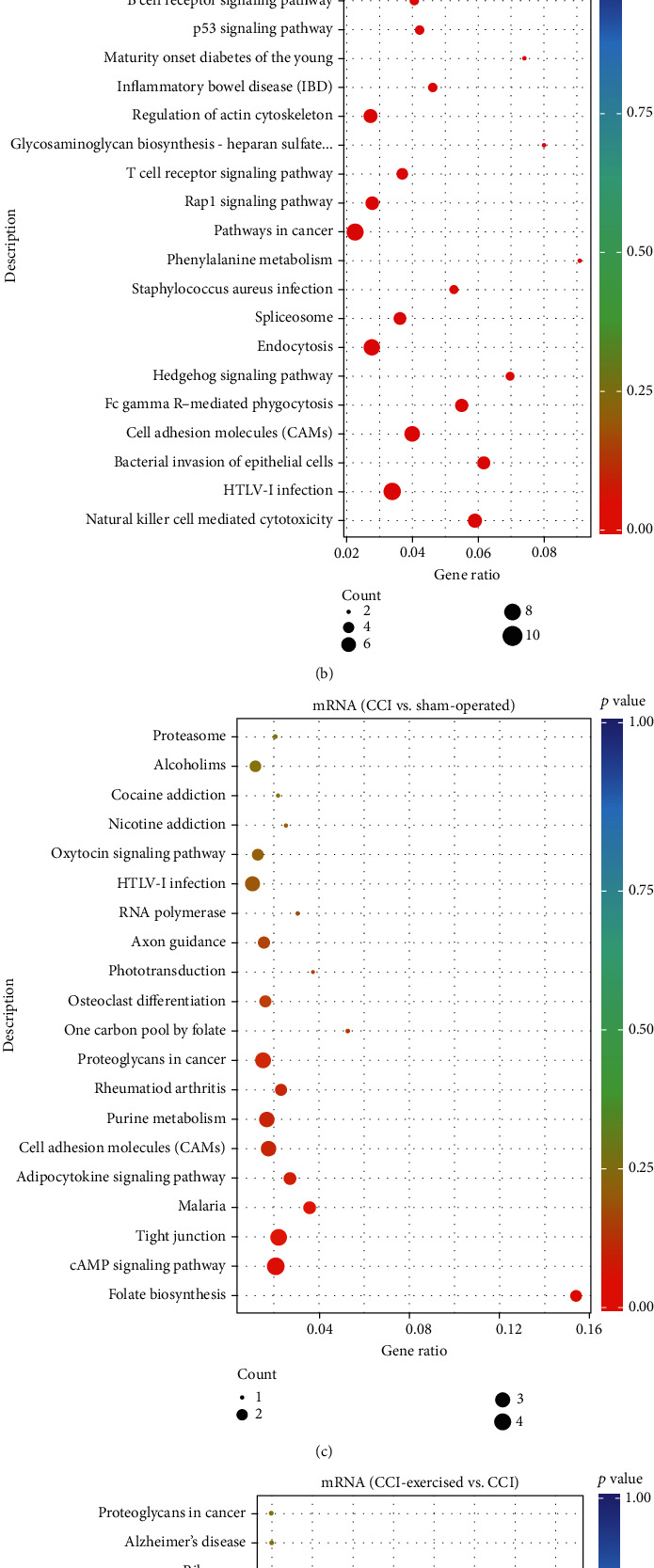
Kyoto Encyclopedia of Genes and Genomes (KEGG) pathway scatterplots of differentially expressed genes (DRGs). MicroRNA- (miRNA-) enriched KEGG pathway scatterplots showing statistics of pathway enrichment in comparisons of (a) the sham-operated group versus the chronic constrictive injury (CCI) group and (b) the CCI-exercised group versus the CCI group. mRNA-enriched KEGG pathway scatterplots showing statistics of pathway enrichment in comparisons of (c) the sham-operated group versus the CCI group and (d) the CCI-exercised group versus the CCI group. The size of the point represents the number of candidate target genes in the pathway, and the color of the point corresponds to different *p* value ranges (*n* = 3 animals per group).

The target gene candidates of differentially expressed miRNAs and differentially expressed mRNAs in the two comparisons (sham-operated group versus CCI group and CCI group versus CCI-exercised group) were subjected to KEGG pathway analysis (Figures 8(a)–8(d)). For miRNAs, 20 pathways were enriched in both comparisons, and the top 5 significantly enriched KEGG pathways included natural killer cell-mediated cytotoxicity, HTLV-I infection, bacterial invasion of epithelial cells, cell adhesion molecules, and Fc gamma R-mediated phagocytosis (*p* < 0.05). Differentially expressed mRNAs were also analyzed, and four signaling pathways were significantly enriched between the CCI and sham-operated groups, including folate biosynthesis, cAMP signaling pathway, tight junction, and malaria (*p* < 0.05). Four pathways were significantly enriched between the CCI and CCI-exercised groups, including HIF-1 signaling pathway, purine metabolism, protein export, and regulation of autophagy (*p* < 0.05).

### 3.6. qRT-PCR Validation of RNA-seq Data

**Figure 9:**
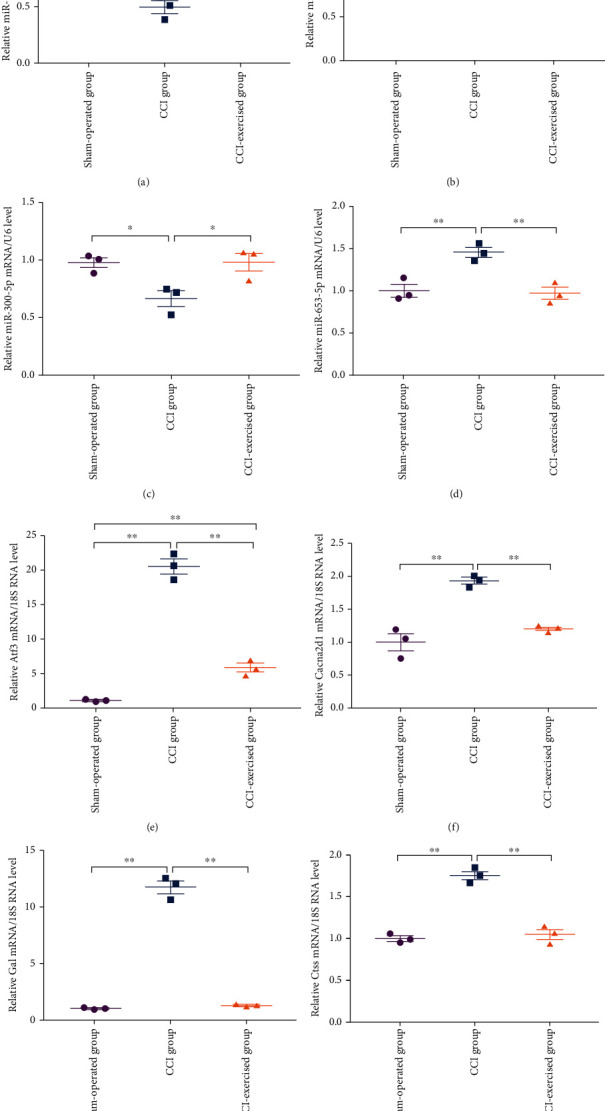
Validation of DEGs by quantitative real-time PCR (qRT-PCR). mRNA expression levels of (a) mir-145-5p, (b) mir-341, (c) mir-300-5p, (d) mir-653-5p, (e) Atf3, (f) Cacna2d1, (g) Gal, and (h) Ctss in the L4–L6 DRG at 28 days after surgery. Analyses of data were done by one-way ANOVA, followed by Tukey's multiple comparison test. Values indicate the mean ± standard error of mean; *n* = 3 animals/group. ∗ denotes *p* < 0.05; ∗∗ denotes *p* < 0.01.

Four differentially expressed miRNAs (miR-145-5p, miR-341, miR-300-5p, and miR-653-5p) and four differentially expressed mRNAs (Atf3, Cacna2d1, Gal, and Ctss) were analyzed by qRT-PCR (Figures 9(a)–9(h)) to confirm the accuracy of the RNA-seq results. All qRT-PCR results were consistent with results obtained from the RNA-seq data.

## 4. Discussion

In this study, the transcriptome responses of NP rats to exercise in the DRG tissue were examined to explore adaptive mechanisms after exercise. Specifically, differentially expressed miRNAs and mRNAs in the L4–L6 DRG were determined by comparisons between CCI, sham-operated, and CCI-exercised rats, after which the functional associations of these DEGs were analyzed. DRG neurons link the peripheral nervous system to the central nervous system, which regulates sensory pathways by receiving nociceptive afferents (e.g., heat, cold, pressure, and chemicals) and then transmitting the information to the spinal dorsal horn [25]. We chose the CCI model, which has been used as a classic model of NP induced by peripheral nerve injury, to simulate the symptoms of chronic sciatic nerve compression in humans [26, 27]. The gene location of miRNA is highly conserved in different species and shows a high degree of homology in sequence [28, 29]. Moreover, the expression patterns of homologous miRNAs have been found to be comparable between organs in human and rat. Here, we want to investigate the miRNAome changes in NP using rats as the animal model and analyze biological functions and pathways of DEGs [30]. Among the differentially expressed miRNAs observed, the expressions of miR-145-5p and miR-300-5p are negatively affected by CCI and positively recovered by exercise. By contrast, miR-653-5p and miR-341 increased after CCI, and these changes could be returned to the control level by exercise. This result suggests that these differentially expressed miRNAs in the DRG may be potential therapeutic goals. Our transcriptome data are consistent with the results of previous studies. For example, Pang et al. studied spinal cord tissue and demonstrated that the expression of miR-145-5p on the 1st, 3rd, 5th, and 7th postoperative days of CCI is significantly decreased compared that observed 1 day before surgery [31]. The genome-wide profile of miRNAs determined in a clinical study indicated that miR-145-5p expression is significantly lower in patients with chronic pain compared with healthy persons [32]. Another study reported that in CCI animal models, overexpression of miR-145-5p was found to significantly improve hyperalgesia induced by mechanical and thermal stimuli [33]. This suggests that miR-145-5p could be involved in exercise to improve the mechanism of CCI-induced hyperalgesia in rats. Using microarray analysis and qRT-PCR analysis, Li et al. revealed that miR-341 expression is significantly upregulated in the DRG of rats with NP compared with that of rats in the normal and sham-operated groups [14].

According to the differentially expressed mRNAs in our transcriptome data, 186 genes in the DRG show altered expression in the both two comparisons of the sham-operated group versus the CCI group and the CCI group versus the CCI-exercised group. Among these differentially expressed mRNAs, according to RGD and previous research, the expressions of Atf3, Ctss, Cacna2d1, and Gal may correlate with NP and contribute to allodynia and hyperalgesia in NP rats. Atf3 is a member of the ATF/cyclic AMP response element-binding transcription factor family and known to be a neuronal injury marker. Previous studies have showed that Atf3 is upregulated in DRG neurons after periphery nerve injury, including CCI, diabetic peripheral neuropathy, and L5 spinal nerve ligation. Moreover, these studies also showed that 4 weeks of exercise training could restore the level of Atf3. [34–37]. Another gene Ctss plays a role in the maintenance of NP. A previous study described that the mRNA expression of lysosomal cysteine protease Ctss in the DRG increased after NP induced by peripheral nerve injury, and inhibitors of Ctss could reverse mechanical allodynia and hyperalgesia [38]. This finding is consistent with our transcriptome data of the comparison between the sham-operated and CCI groups. Ctss has been found to be expressed in microglia in the spinal cord and brain after NP [39]. Thus, the elevated expression of Ctss in the peripheral and central nervous systems may contribute to NP of CCI rats. Cacna2d1 is expressed in neurons throughout the central nervous system, with enrichment in the DRG, spinal dorsal horn, and cortical and hippocampal neurons [40]. Previous studies reported that nerve injury increases the expression of Cacna2d1 [21, 41–43]. Furthermore, Cacna2d1 is a voltage-gated calcium channel subunit, and its dysregulation may contribute to NP states by modulating synaptic transmission and plasticity, such as abnormal synaptogenesis [44–46]. Such changes can alter the activities of neuronal networks that fundamentally contribute to the cellular basis of NP development [47]. The Gal neuropeptide shows neuroprotection against the damage induced by nerve injury. Xu et al. [48] found that the expression levels of Gal and its receptors GalR1 and GalR2 were significantly increased in both DRG and spinal dorsal horn of bilateral CCI rats and decreased after intrathecal injection of exogenous Gal. This suggests that Gal may participate in reducing NP by activating GalR1 and GalR2 receptors. These studies suggest that Atf3, Ctss, and Cacna2d1 are pain inducers, and Gal was a pain protector. Previous studies and our transcriptome data confirmed that Atf3, Ctss, Cacna2d1, and Gal are significant upregulated after NP. Moreover, the expressions of Atf3, Ctss, Cacna2d1, and Gal were recovered after 4 weeks of exercise training, which may lead to improvements in mechanical allodynia and hyperalgesia in exercised CCI rats.

GO analysis revealed that signal transduction, defense response, and calcium channel activity are the main responses to peripheral nerve injury between the sham-operated and CCI groups. Additionally, between the CCI and CCI-exercised groups, defense response, voltage-gated calcium channel complex, kinase activator activity, postsynaptic endocytic zone, and methyltransferase activity were the main responses to exercise after CCI in DRG neurons. KEGG analysis revealed that the DEGs were significantly enriched in the classifications of Inflammatory mediator regulation of TRP channels, TNF signaling pathway, Rap1 signaling pathway, NF-kappa B signaling pathway, and MAPK signaling pathway in the comparison of the Sham-operated group versus the CCI group. After 4 weeks of exercise, the HIF-1 signaling pathway, Rap1 signaling pathway, T cell receptor signaling, B cell receptor signaling pathway, and neurotrophin signaling pathway were enriched. It is found that these pathways are closely related to the occurrence and development of NP through retrieving. For example, the TNF signaling pathway was significantly enriched in the sham-operated group compared with the CCI group. TNF superfamily cytokines represented a group of multifunctional proinflammatory cytokines that activated the signaling pathways of cell survival, apoptosis, inflammatory response, and cell differentiation. There is increasing evidence that TNF-*α* plays an important role in NP. Xu et al. showed that the injury of L5 nerve root transection can cause persistent mechanical allodynia and hyperalgesia in the hind paw of rats [49]. The immunoreactivity of TNF-*α* and its receptor 1 (TNFR1) in the damaged DRG increased significantly from 1 day after injury and lasted for 2 weeks. Immunofluorescence double staining showed the increase of TNF-*α* in satellite glia, microglia, and neurons, while TNFR1 only existed in DRG neurons. Preoperative intraperitoneal injection of TNF-*α* inhibitor can block mechanical allodynia and hyperalgesia. However, if it was used on the 7th day after the operation, the pain could not be reversed. These data suggest that the upregulation of TNF-*α* and TNFR1 in DRG and spinal dorsal horn is necessary for the initiation of NP caused by L5 nerve root transection, but they are not the key factors for subsequent pain maintenance [49]. Furthermore, the neurotrophin signaling pathway was enriched in the comparison of the CCI group versus the CCI-exercised group. The neurotrophins are composed of nerve growth factor (NGF), brain derived neurotrophic factor (BDNF), neurotrophin-3 (NT-3), and neurotrophin-4/5 (NT-4/5), which mediated by the neurotrophin receptor p75NTR and the tropomyosin receptor kinases (Trks) [50]. Atf3 has been found to be positive in 82% of L4 DRG neurons after sciatic nerve injury, but the positive rate decreased to 35% after the intrathecal delivery of NGF [51]. These results demonstrate that the expression of Atf3 may be induced by loss of NGF. Moreover, Geremia et al. [52] revealed that electrical stimulation increased BDNF expression in DRG neurons following peripheral nerve injury. The application of neurotrophins in NP shows that neurotrophins play an important role in peripheral nerve repair.

There are some limitations in this study. Firstly, the time point of 4 weeks after exercise was chosen on the basis of previous studies to observe expression changes of genes in response to exercise. However, in the process of behavioral analysis, it was found that mechanical hyperalgesia and thermal hyperalgesia were also significantly improved at 3 weeks. Therefore, the present study does not sufficiently reflect all important alterations in the DRG transcriptome after exercise. We recommend further research with more time points to address this limitation. Secondly, only male rats were used, and the potential sex dimorphic results are not reflected in our study. Estrogen can influence pain behaviors in rats [53]. Fan et al. [54] reported that the change of androgen level has no obvious effect on pain threshold, while ovarian hormone may inhibit the formation of mechanical hyperalgesia, but it had no obvious effect on the thermal pain threshold. We need to pay more attention to females in future research.

## 5. Conclusion

This study provides an understanding of the adaptive mechanisms after exercise of the DRG by transcriptional profiling. Multiple DEGs (such as miR-145-5p, miR-341, miR-300-5p, miR-653-5p, Atf3, Cacna2d1, Gal, and Ctss) were identified under pairwise comparisons of the sham-operated group versus the CCI group and the CCI group versus the CCI-exercised group. The differentially expressed miRNAs and mRNAs identified in this work may be new therapeutic targets for the treatment of NP induced by peripheral nerve injury. However, further miRNA interference experiments need to be carried out to confirm whether the target genes will change with the increase or decrease of expression of these differentially expressed miRNAs.

## Figures and Tables

**Table 1:** The primers used in qRT-PCR.

Gene	5′ to 3′
U6 forward	CGCAAGGATGACACGCAAATTCG
mir-145-5p forward	CAGTTTTCCCAGGAATCCCT
miR-653-5p forward	TTGAAACATTCTCTACTGAAC
miR-341 forward	GTCGATCGGTCGGTCGGT
miR-300-5p forward	TTGAAGAGAGGTTATCCTTTGT
Universal miRNA/U6 reverse primer	Provided by the reagent kit
Atf3 forward	GCCATCCAGAACAAGCACC
Atf3 reverse	CACTTGGCAGCAGCAATTT
Gal forward	GGTTCCCACCACTGCTCAAG
Gal reverse	CCTTTGTTGGCATCCCGAG
Cacna2d1 forward	GGCGTTGATGTGTCTCTGGA
Cacna2d1 reverse	AGTGTGACCGGCTCCTTTG
Ctss forward	TGGTGGATTGCTCAACCGAA
Ctss reverse	TCAGAGCTTCTTCATCGCCG
18 s rRNA forward	GCTTAATTTGACTCAACACGGGA
18 s rRNA reverse	AGCTATCAATCTGTCAATCCTGTC

**Table 2:** Comparison of differentially expressed mRNAs with NP-related phenotypes in RGD database.

Gene	Comparison1 *p* value	Comparison1 log_2_ FC	Comparison2 *p* value	Comparison2 log_2_ FC	NSD	Neuralgia	Pain	Hyperalgesia	Inflammation
Atf3	3.20*E* − 21	4.045819	0.001948	-1.442530	√				
Gal	7.18*E* − 11	3.829123	0.003094	-2.022188		√		√	√
Cacna2d1	4.61*E* − 08	1.261635	0.014522	-0.607313		√	√	√	
Nos1	3.00*E* − 07	1.799434	0.030345	-0.835820	√	√		√	
Reg3b	3.49*E* − 07	4.162273	0.004735	-2.012063	√				
Adcyap1	6.55*E* − 06	1.235189	0.019065	-0.639252				√	
Gch1	6.40*E* − 05	1.984458	0.047527	-0.959824			√		
Sdc1	7.90*E* − 05	2.078746	0.013891	-1.300921					√
Il1a	0.000680	7.930640	0.000627	-8.012905				√	√
Tnfaip6	0.000696	2.455342	0.003988	-2.044203					√
Ctss	0.004446	0.836964	0.017493	-0.774995	√	√		√	
Ltf	0.010002	7.393181	0.009157	-7.477871					√
Spp1	0.010235	0.605711	0.036589	-0.494265	√				√
Cyp11a1	0.026784	-7.823132	0.004030	8.228974	√				

Comparison1 represents the sham-operated group vs. CCI group. Comparison2 represents the CCI group vs. CC-exercised group; FC: fold change; NDS: nervous system disease.

## Data Availability

The datasets generated for this study have been deposited in the Sequence Read Archive database of NCBI, and the BioProject accession is PRJNA734377.

## Supplementary Materials

Supplementary Materials Table S1: sequencing data quality summary of microRNAs (miRNAs). Table S2: sequencing data quality summary of mRNAs.
