# Supplementary material for: Comparative Transcriptome Profiling Reveals Changes of microRNAs Response to Exercise in Rats with Neuropathic Pain
Source: Neural Plast. 2021 Aug 2;2021:5597139. doi: 10.1155/2021/5597139 (PMC8356008; doi:10.1155/2021/5597139)
Supplement: Supplementary Materials — Table S1: sequencing data quality summary of microRNAs (miRNAs). Table S2: sequencing data quality summary of mRNAs. [file 5597139.f1.zip › Table S1.docx]

**Table S1** **Sequencing data quality summary of miRNAs**

| Sample name | Raw reads | Clean reads | Bases | Error rate (%) | Q20 (%) | Q30(%) | GC content（%） |
| --- | --- | --- | --- | --- | --- | --- | --- |
| Sham_1 | 18647987 | 17543614 | 0.932G | 0.01 | 97.11 | 91.63 | 49.91 |
| Sham_2 | 16292348 | 15994175 | 0.815G | 0.01 | 97.23 | 91.98 | 49.50 |
| Sham_3 | 15271271 | 15016838 | 0.764G | 0.01 | 97.56 | 92.58 | 49.46 |
| CCI_1 | 14394485 | 13879131 | 0.720G | 0.01 | 97.72 | 92.93 | 49.43 |
| CCI_2 | 12579150 | 12141494 | 0.629G | 0.01 | 97.52 | 92.38 | 49.79 |
| CCI_3 | 13659287 | 13194011 | 0.683G | 0.01 | 97.81 | 93.22 | 49.18 |
| Swim_1 | 14122533 | 13603295 | 0.706G | 0.01 | 97.81 | 93.04 | 49.48 |
| Swim_2 | 13193737 | 12727005 | 0.660G | 0.01 | 97.68 | 92.77 | 49.43 |
| Swim_3 | 15498247 | 14868928 | 0.775G | 0.01 | 97.57 | 92.75 | 49.40 |
