# Supplementary material for: Comparative Transcriptome Profiling Reveals Changes of microRNAs Response to Exercise in Rats with Neuropathic Pain
Source: Neural Plast. 2021 Aug 2;2021:5597139. doi: 10.1155/2021/5597139 (PMC8356008; doi:10.1155/2021/5597139)
Supplement: Supplementary Materials — Table S1: sequencing data quality summary of microRNAs (miRNAs). Table S2: sequencing data quality summary of mRNAs. [file 5597139.f1.zip › Table S2 .docx]

**Table S2 Sequencing data quality summary of mRNAs**

| Sample name | Raw reads | Clean reads | Raw bases | Clean bases | Error rate (%) | Q20 (%) | Q30 (%) | GC content（%） |
| --- | --- | --- | --- | --- | --- | --- | --- | --- |
| Sham_1 | 102769296 | 100576230 | 15.42 | 15.09 | 0.03 | 97.20 | 92.42 | 47.87 |
| Sham_2 | 96894488 | 94730716 | 14.53 | 14.21 | 0.03 | 97.24 | 92.49 | 47.93 |
| Sham_3 | 107206228 | 104573894 | 16.08 | 15.69 | 0.03 | 97.43 | 92.95 | 48.18 |
| CCI_1 | 86083972 | 78808508 | 12.91 | 11.82 | 0.03 | 96.87 | 91.91 | 52.47 |
| CCI_2 | 103320270 | 101026472 | 15.50 | 15.15 | 0.03 | 97.15 | 92.40 | 48.21 |
| CCI_3 | 121559936 | 117271708 | 18.23 | 17.59 | 0.03 | 97.44 | 92.91 | 49.01 |
| Swim_1 | 123191458 | 120440852 | 18.48 | 18.07 | 0.03 | 97.47 | 92.97 | 48.11 |
| Swim_2 | 108203730 | 105607538 | 16.23 | 15.84 | 0.03 | 96.77 | 91.50 | 47.93 |
| Swim_3 | 104885688 | 102609940 | 15.73 | 15.39 | 0.03 | 97.32 | 92.63 | 48.31 |
